# Octopaminergic modulation of contrast sensitivity

**DOI:** 10.3389/fnint.2012.00055

**Published:** 2012-08-03

**Authors:** Roel de Haan, Yu-Jen Lee, Karin Nordström

**Affiliations:** Department of Neuroscience, Uppsala UniversityUppsala, Sweden

**Keywords:** motion adaptation, activity state, CDM, contrast gain reduction, motion vision, insect vision, intracellular electrophysiology, input resistance

## Abstract

Sensory systems adapt to prolonged stimulation by decreasing their response to continuous stimuli. Whereas visual motion adaptation has traditionally been studied in immobilized animals, recent work indicates that the animal's behavioral state influences the response properties of higher-order motion vision-sensitive neurons. During insect flight octopamine is released, and pharmacological octopaminergic activation can induce a fictive locomotor state. In the insect optic ganglia, lobula plate tangential cells (LPTCs) spatially pool input from local elementary motion detectors (EMDs) that correlate luminosity changes from two spatially discrete inputs after delaying the signal from one. The LPTC velocity optimum thereby depends on the spatial separation of the inputs and on the EMD's delay properties. Recently it was shown that behavioral activity increases the LPTC velocity optimum, with modeling suggesting this to originate in the EMD's temporal delay filters. However, behavior induces an additional post-EMD effect: the LPTC membrane conductance increases in flying flies. To physiologically investigate the degree to which activity causes presynaptic and postsynaptic effects, we conducted intracellular recordings of *Eristalis* horizontal system (HS) neurons. We constructed contrast response functions before and after adaptation at different temporal frequencies, with and without the octopamine receptor agonist chlordimeform (CDM). We extracted three motion adaptation components, where two are likely to be generated presynaptically of the LPTCs, and one within them. We found that CDM affected the early, EMD-associated contrast gain reduction, temporal frequency dependently. However, a CDM-induced change of the HS membrane conductance disappeared during and after visual stimulation. This suggests that physical activity mainly affects motion adaptation presynaptically of LPTCs, whereas post-EMD effects have a minimal effect.

## Introduction

Sensory systems provide physiologically relevant representations of the surrounding world (Atick, 1992; Rieke et al., 1995; Simoncelli and Olshausen, 2001; Field and Chichilnisky, 2007). The sensory world is highly complex and diverse, and can contain an almost infinite number of possible inputs that must be coded by a fixed number of neurons with limited bandwidth (see e.g., Atick, 1992). Reliable and sensitive coding of sensory input is important for survival, since precise and detailed knowledge of the world is needed to maximize fitness and to reproduce, by e.g., smelling food when one passes it, hearing an approaching predator, or identifying a potential mate. To be able to code these broad inputs, and to enable signaling of even small deviations, each neuron adapts to the currently prevailing stimulus conditions (Maddess and Laughlin, 1985; Ulanovsky et al., 2003; Kurtz et al., 2009a). The process of such neural adaptation is well studied in a number of systems, ranging from primate cortical MT neurons (Kohn and Movshon, 2003), through the cat visual (Hu et al., 2011) and auditory cortex (Ulanovsky et al., 2003), and the visual inter-neurons of the fly lobula plate (Maddess and Laughlin, 1985; Harris et al., 2000; Fairhall et al., 2001; Neri and Laughlin, 2005; Kalb et al., 2008; Kurtz et al., 2009a). These studies show that adaptation changes the neural coding range to code the distribution of stimuli that is being encountered, not only by shifting the sensitivity range to the current mean stimulus and reducing the output to a continuous stimulus (Maddess and Laughlin, 1985; Kurtz et al., 2009a), but also by adjusting the coding sensitivity to the spread of the stimulus (Fairhall et al., 2001; Ulanovsky et al., 2003).

Adaptation to prolonged visual motion in flies has been particularly well investigated. This has typically been done using electrophysiology of single neurons in the fly optic lobes of immobilized animals (e.g., Maddess and Laughlin, 1985; Harris et al., 2000; Fairhall et al., 2001; Neri and Laughlin, 2005). Recently, however, a clear disadvantage of this experimental protocol has come to light: new experimental techniques show that the responses of fly motion-sensitive neurons change according to the behavioral state of the animal (e.g., Chiappe et al., 2010; Maimon et al., 2010; Rosner et al., 2010; Jung et al., 2011). Changing the coding of motion according to the animal's behavioral state is neuroethologically sound since lobula plate tangential cells (LPTCs) respond to widefield motion, such as that generated by ego-motion (see e.g., Franz and Krapp, 2000; Karmeier et al., 2006; Borst et al., 2010). Recent work has highlighted that LPTCs serves quite a complex role in motion vision: horizontal system (HS) neurons respond strongly not only to yaw rotation, as suggested by their receptive fields (Krapp et al., 2001), but also to self-induced translation (Boeddeker et al., 2005), and to individual, salient features within background optic flow (O'Carroll et al., 2011; Liang et al., 2012).

Since LPTCs serve a role in active vision, they could be expected to respond differently in physically active animals compared with resting animals, especially considering that neural transmission is metabolically expensive (Laughlin et al., 1998; Attwell and Laughlin, 2001; Laughlin, 2001; Lennie, 2003; Niven and Laughlin, 2008). Therefore, different modes of motion adaptation may exist, one while the animal is stationary, mainly reducing energetic costs of continuous neural signaling, the other while the animal is moving, giving it high accuracy to manoeuvre through the complex visual world.

LPTCs spatially pool input from many elementary motion detectors (EMDs). EMDs correlate the luminance change from two spatially separated photo-inputs after delaying the signal from one. By subtracting the output from a mirror symmetric subunit, direction selectivity is generated (Borst and Euler, 2011). Several authors have suggested that adaptation may shorten the time constant of the EMD's delay filter (e.g., de Ruyter van Steveninck et al., 1986; Clifford et al., 1997), which should shift the adapted velocity optimum to higher velocities (Harris et al., 1999). This shift is physiologically relevant only in physically active animals, since a resting animal does not generate optic flow. Indeed, a shift to a higher velocity optimum was not found after adaptation in immobilized flies (Harris et al., 1999), whereas it was observed when flies were able to move (Chiappe et al., 2010; Jung et al., 2011). Recording from physically active animals is technically challenging (Chiappe et al., 2010; Maimon et al., 2010; Rosner et al., 2010), but the effects of locomotion can be mimicked by application of the octopamine receptor agonist chlordimeform (CDM, see e.g., Longden and Krapp, 2009; Jung et al., 2011). Octopamine is the insect equivalent of the mammalian adrenergic transmitters, triggering the fight-or-flight response and increasing metabolism (Roeder, 2005). It is released throughout the hemolymph during insect flight (Goosey and Candy, 1980).

Modeling suggests that the observed velocity optimum shift caused by CDM or physical activity may be generated presynaptically of the LPTCs, by altering the time-constants of different temporal filters within the EMD (Jung et al., 2011). However, another recent paper showed another physiological postsynaptic effect: the conductance of *Drosophila* LPTCs themselves also changes with the fly's locomotor state (Maimon et al., 2010). It is therefore unclear to which extent effects within the EMD (presynaptic to the LPTCs) and within the LPTCs themselves (postsynaptic to the EMD) contribute to the observed physiological changes of LPTCs caused by physical activity, or CDM application.

Visual motion adaptation can be separated into different components, where two are likely to be generated presynaptically of the LPTCs, and two within these neurons (Nordström and O'Carroll, 2009; Nordström et al., 2011). The components can be extracted by producing contrast response functions before and after adaptation (Harris et al., 2000): (1) The after-potential generates a vertical shift of the adapted contrast response function. Since the after-potential is direction-selective (Harris et al., 2000) it is likely generated after the summation stage of the EMD, which probably takes place in the input dendrites to the LPTCs (e.g., Single et al., 1997). An LPTC origin is supported by the observation that the after-potential is global, meaning that when one part of a neuron's receptive field is adapted, the after-potential is present in previously un-stimulated parts of the receptive field (Nordström and O'Carroll, 2009). The exact mechanism by which the after-potential arises is not completely clear, although evidence hints at an activity dependent inhibitory conductance (Kurtz et al., 2000), activated by intracellular Na^+^ or another intracellular messenger signaling activity, other than Ca^2+^ (Kurtz, 2007). (2) The non-directional contrast gain reduction generates a right-shift of the adapted contrast response function. Contrast gain reduction is also mildly recruited by flicker adaptation and is visible after as little as 20 ms of motion adaptation (Harris et al., 2000; Nordström et al., 2011). Since the effect is non-directional it is likely to be recruited before the summation stage of the EMD. A presynaptic mechanism is supported by the observation that the contrast gain reduction is local, affecting only those input dendrites that have been directly subjected to adaptation (Nordström and O'Carroll, 2009). (3) The output range reduction is also local and non-directional, and therefore likely generated presynaptically to the LPTC. It is not recruited by flicker (Harris et al., 2000) and has a slower onset than contrast gain reduction (Nordström et al., 2011), suggesting that different cellular mechanisms underlie contrast gain and output range reduction.

To physiologically separate the CDM-induced effects on motion adaptation into their EMD and post-EMD components, we used test-adapt-test protocols together with pharmacological activation of octopamine receptors. We show that the contrast gain reduction changes when CDM is applied, from being temporal frequency independent to being frequency dependent, while leaving two other components of motion adaptation unchanged. Furthermore, we investigated the power spectral density of the membrane potential and show that CDM changes the input resistance of LPTCs when there is no visual stimulation, but that this effect disappears during and after stimulation, suggesting that it is unlikely to contribute to the effects seen on motion adaptation and velocity tuning.

## Materials and methods

### Electrophysiology

Male hoverflies (*Eristalis* spp.) were reared from larvae gathered at cow farms near Uppsala University. After pupation and hatching the flies were kept in a net (~2.5 m^3^) under a 12 h light/dark cycle at 22–25°C. The flies were allowed *ad libitum* access to a mixture of pollen, sugar, and water.

At experimental time the hoverfly was immobilized in an eppendorf tube. A relatively large hole was cut over the left lobula complex, to be able to carefully add solutions without the surface tension moving the electrode. Experiments were carried out at 21–24°C. The signal was amplified using a BA-03X amplifier (npi electronic, Germany), with 50 Hz electric noise eliminated with a Hum Bug (Quest Scientific, Canada). The signal was digitized at 10 kHz using a Powerlab 4/30 and visualized and recorded with LabChart software (both AD Instruments, Australia).

Horizontal system north (HSN) neurons in the left lobula plate were recorded intracellularly by impaling them with a sharp aluminosilicate micropipette pulled on Sutter Instruments P-1000 and filled with 2 M KCl. Electrodes had a resistance of 85–220 MΩ. HSN neurons were identified based on the receptive field and directional selectivity (Nordström et al., 2008).

### Visual stimulation

The fly was mounted facing the center of an RGB CRT monitor with a frame rate of 160 Hz, a mean illuminance of 135 lx and a spatial resolution of 640 × 480 pixels, corresponding to ca. 100 × 75° of the fly's visual field of view. Stimuli were generated with custom software (http://www.flyfly.se) using the psychophysics toolbox (http://psychtoolbox.org) in MATLAB (The MathWorks, USA).

**Figure 1:**
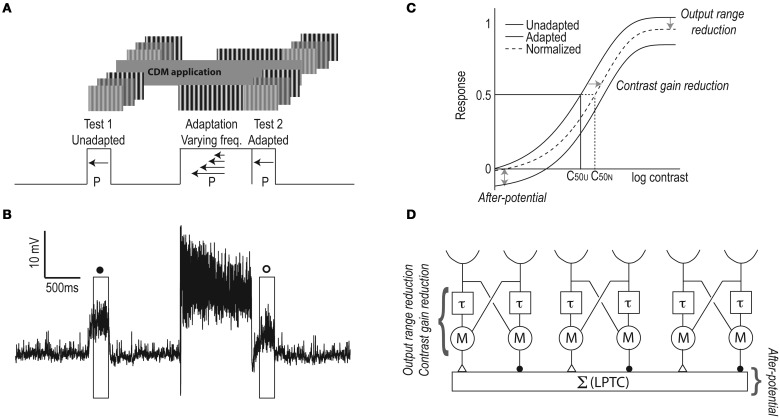
**The test-adapt-test protocol. (A)** A drifting stimulus grating (0.1 cpd, 5 Hz) of varying contrast was shown for 300 ms (Test 1), followed by 1 s of mid-luminance screen, 1 s adapting stimulus (0.1 cpd, moving at 2.5, 7.5, 12.5, or 20 Hz) at 0.95 contrast, immediately followed by a 300 ms second test stimulus (Test 2) with parameters identical to the first test. All sinusoidal gratings drifted in the preferred direction (P) of the neuron. After adapting at four temporal frequencies, we applied CDM and repeated the protocol. **(B)** A typical depolarizing response of a male HSN, with the test contrast at 0.08, and the adapting stimulus at 12.5 Hz. Boxes indicate the analysis windows: open symbols represent adapted responses and filled symbols un-adapted responses, as throughout the paper. **(C)** Three components of motion adaptation (as identified by Harris et al., 2000). The antagonistic after-potential gives a vertical shift of the contrast sensitivity function (up or down). Contrast gain reduction gives a right-shift of the adapted contrast response function, even after subtraction of the after-potential. Output range reduction gives a compression of the response to high-contrast stimuli in the adapted neuron. C_50U_ and C_50N_ indicate the un-adapted and normalized C_50_ values (i.e., the contrast that generates 50% maximum response in the un-adapted neuron), respectively. **(D)** A graphical representation of EMDs feeding into an LPTC (there are obviously more then three EMDs supplying input to each LPTC). Each EMD receives input from two spatially separated ommatidia (semi-circles). The input from one of these goes through a neural delay (τ), which is then multiplied (M) with the un-delayed input from a neighboring input. In the summation stage (Σ) the inputs from mirror symmetric subunits are subtracted (inhibitory synapses illustrated as filled circles, excitatory synapses as open triangles), thus generating direction selective responses in the LPTC.

We used test-adapt-test protocols adapted from Harris et al. (2000) with full-screen sinusoidal test gratings (5 Hz, 0.1 cpd) of different contrasts, and adapting gratings (2.5, 7.5, 12.5, or 20 Hz, at 0.1 cpd) with a contrast of 0.95, with the screen calibrated to give a linear relationship between input RGB values and output illuminance (Hagner Luxmeter E2). Between the first test stimulus and the adapting stimulus the screen was at mean-luminance for 1 s, and the second test stimulus immediately followed the adapting stimulus (Figure 1A). The test stimuli were displayed for 300 ms (Harris et al., 2000), and adapting stimuli for 1 s. Earlier work on motion adaptation used longer adaptation times (e.g., Maddess and Laughlin, 1985; Harris et al., 2000; Borst et al., 2005; Neri and Laughlin, 2005; Kurtz et al., 2009a). However, we recently showed that whereas the strength of the motion adaptation components increases with the adapting duration, all components are significant after as little as 500 ms of adaptation (Nordström et al., 2011). To optimize the experimental protocol, we therefore adapted for 1 s. Between trials the screen was left at mean-luminance, for a minimum of 3 s. All stimuli moved in the preferred direction at velocities in the physiologically relevant range of *Eristalis* (Straw et al., 2006).

Weekly a stock solution of CDM (Sigma Aldrich, Germany) was made by dissolving CDM in Ringer's solution (Karmeier et al., 2001) at a concentration of 50 μM, and stored at 4°C. On the day of use the CDM solution was diluted to 10 μM. After a first set of test-adapt-test protocols, taking approximately 20 min, 2.5 μl of the octopamine receptor agonist CDM was carefully applied with a micropipette. This amount and concentration ensured that the droplet could be completely absorbed into the head capsule, while keeping the total amount of CDM as high as the amount that gave a clear response in Longden and Krapp (2010). As CDM is tissue permeable this gives a similar effect, but the smaller droplet is absorbed more quickly into the head capsule area, thus reducing the diffusion time. After application, CDM was allowed to dissolve throughout the hoverfly's head capsule for five minutes, while monitoring recording stability.

### Data analysis

Data were analyzed in MATLAB. Repetitions within each neuron were averaged before averaging over different animals (N). For each fly we recorded responses to 1–4 repetitions (n) at each test contrast, before and after adapting at four different temporal frequencies, in two pharmacological conditions (without and with CDM). We only kept data from flies where we completed the entire protocol.

Responses to test stimuli were defined as the mean membrane potential between 100 and 300 ms post-stimulus onset [Figure 1B, as in previous work, see e.g., Harris et al. (2000)]. A Weibull function was fitted to the data to describe the relationship between test-stimulus contrast and neural response:

$$f(x)=\text{offset}+\text{gain}(1-e^{-{\left(x/\alpha \right)}^{\beta }})$$

where *f(x)* is the response to a test stimulus with contrast *x*, the *offset* is the lower value at which the function asymptotes, and the *gain* the value at which the function saturates. α and β define the scaling and the steepness of the function, respectively. All parameters were free to vary while fitting. The function was fitted to each data set using a simplex search (Lagarias et al., 1998).

From the resulting contrast response functions we extracted three components of motion adaptation (Figure 1C, and see Harris et al., 2000): (1) The antagonistic (i.e., direction-selective) after-potential was defined as the response to the 0 contrast test stimulus. We normalized the data by subtracting the after-potential from the adapted responses (as in Harris et al., 2000). (2) The direction independent contrast gain reduction generates a rightward shift of the adapted contrast response function. C_50_ was extracted from the Weibull fits, and defined as the contrast that generates a 50% maximum response in the un-adapted neuron (Figure 1C). We quantified the C_50_ increase by dividing the normalized C_50_ by the un-adapted C_50_. (3) The output range reduction was quantified as the normalized response to a test contrast of 1.0.

Power spectra were computed using a fast Fourier transform (FFT) of the raw membrane potential. We investigated the effect of stimulation and adaptation on the increased membrane fluctuations by taking the power spectral density of the recorded membrane potential during 100–300 ms post-test stimulus onset, when the screen was at mean-luminance (contrast = 0), and when the test stimuli were at full contrast. We quantified the mean power between 50 and 150 Hz.

Response latency was calculated by first determining the standard deviation (SD) of the pre-stimulus membrane potential (Warzecha and Egelhaaf, 2000), and then identifying the time point at which the response passed 2 × SD post-stimulus onset. This could only be determined in the unadapted neurons, since the adapted responses immediately followed adapting stimuli (Figure 1A), making it impossible to determine adapted response delays.

Paired *t*-tests or Two-Way repeated measures ANOVAs, where necessary followed by a Holm-Bonferroni correction, were done to indicate significance. The data showed a normal distribution (D'Agostino-Pearson omnibus K2 test). Statistical significant difference was allocated to *p* < 0.05. Error bars in figures show standard error of the mean (SEM). Numbers given in the text refer to mean ± SD.

## Results

### CDM interacts with adaptation through the contrast gain reduction

**Figure 2:**
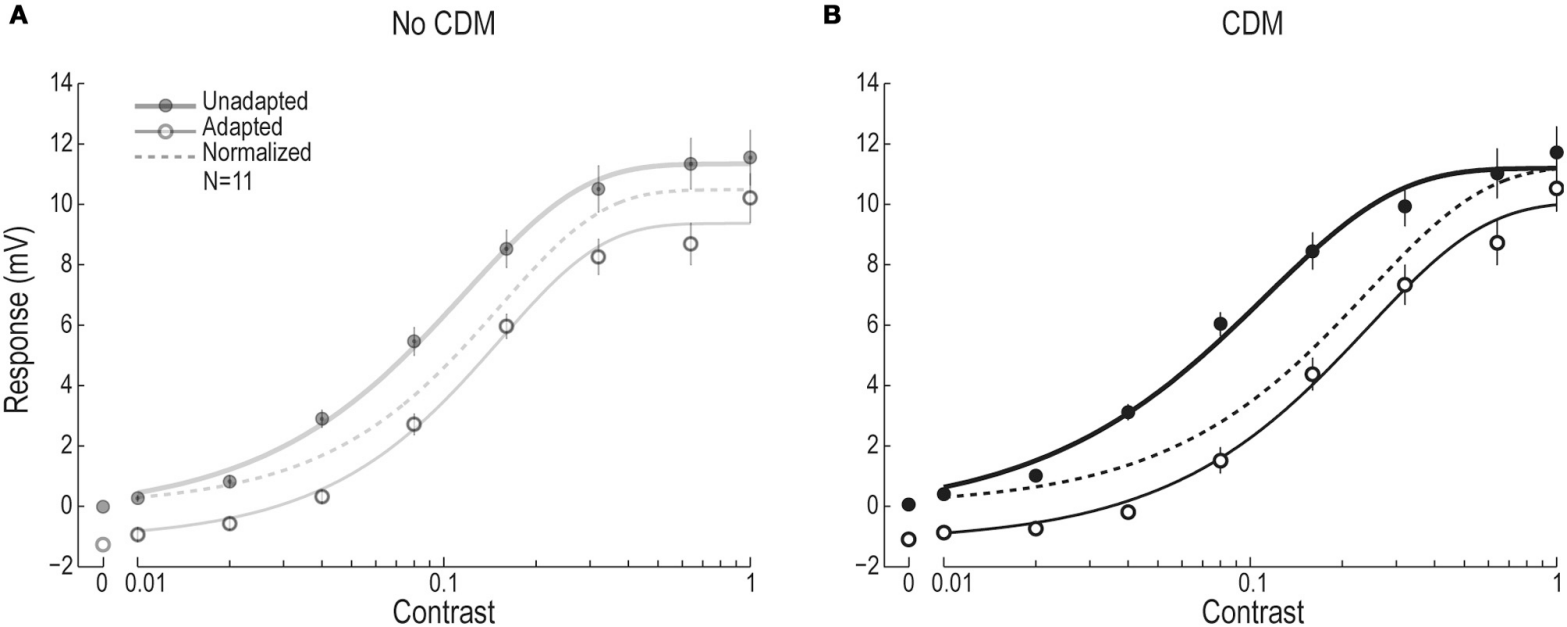
**Contrast response function before and after 20 Hz adaptation, with and without CDM. (A)** The un-adapted (filled symbols) and adapted (open symbols) contrast response function of male HSN, with the adapting stimulus moving at 20 Hz (full-screen sinusoidal grating, 0.1 cpd, contrast = 0.95, *N* = 11). The solid lines show the fitted Weibull functions and the dashed line the adapted function after subtraction of the after-potential. **(B)** The contrast response function before and after adapting at 20 Hz, in the presence of CDM (*N* = 11).

To investigate the presynaptic and postsynaptic contributions to the previously reported CDM-induced, temporal frequency dependent effect on visual motion adaptation (Longden and Krapp, 2010; Jung et al., 2011), we used test-adapt-test protocols in which we varied the contrast of the test pattern and adapted at four different temporal frequencies (Figure 1A). The resulting contrast response functions allow us to extract three different components of motion adaptation (Harris et al., 2000), two of which are generated presynaptically, and one postsynaptically (Figures 1C,D). After adapting at 20 Hz for 1 s (open symbols, Figure 2A) we see a contrast gain reduction, as in earlier work (Nordström et al., 2011). The contrast gain reduction is present even after normalizing the adapted data, by subtracting the hyperpolarizing after-potential (dashed line, Figure 2A). After application of CDM, the contrast gain reduction appears to have increased, by producing a larger rightward shift of the adapted contrast response function (Figure 2B). The un-adapted C_50_, however, does not change (C_50Pre_ = 0.081 ± 0.018; C_50CDM_ = 0.089 ± 0.016).

**Figure 3:**
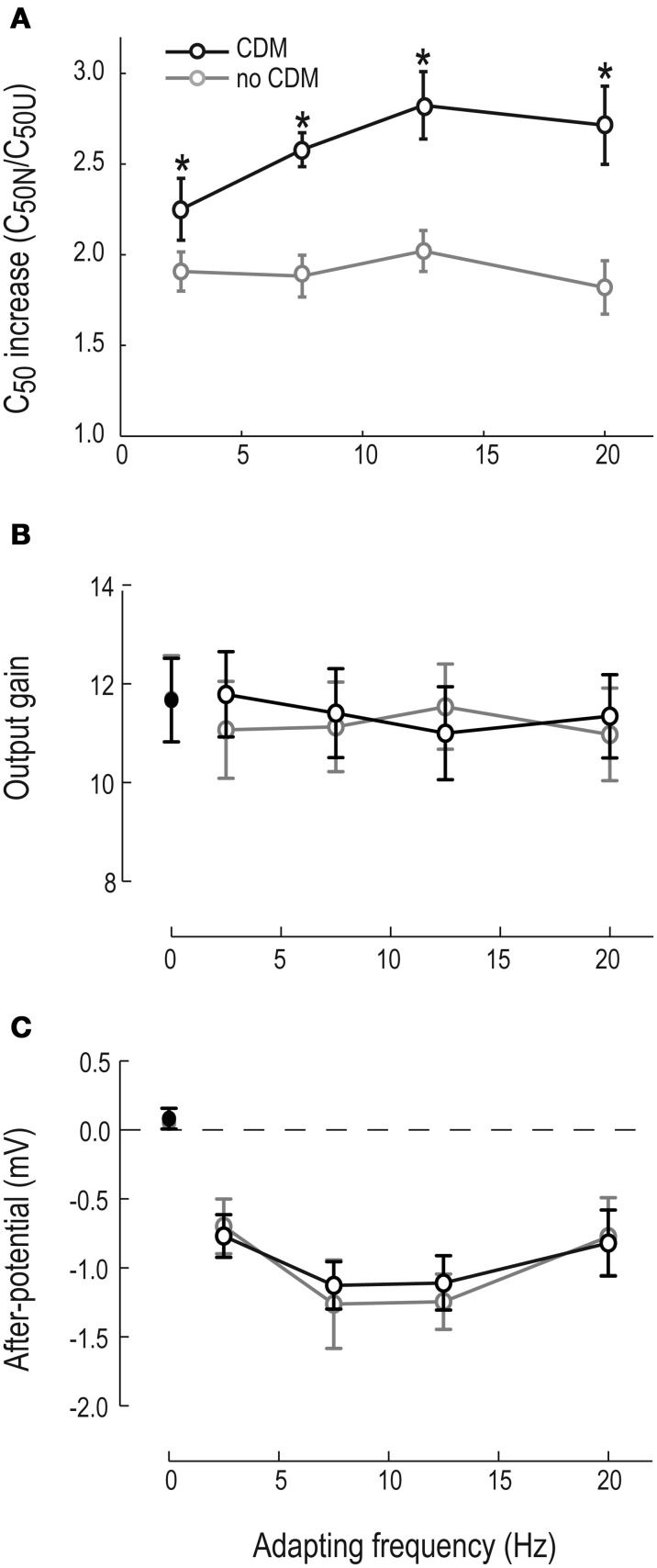
**Motion adaptation components, with and without CDM. (A)** The C_50_ increase (i.e., C_50*normalized*_/C_50*un-adapted*_) as a function of adapting frequency. Asterisks (^*^) indicate significant difference between pre-CDM and post-CDM, with significance allocated to *p* < 0.05 (paired *t*-tests). Un-adapted contrast gain is not shown, since using our definition it equals 1. **(B)** Output gain as a function of adapting frequency. The un-adapted output gain is shown as 0 on the x-axis. **(C)** The after-potential as a function of adapting frequency. The un-adapted “after-potential” is shown inset as 0 on the x-axis. *N* = 11 in all panels.

We quantify the contrast gain reduction by dividing the normalized C_50_ with the unadapted C_50_ (C_50N_ and C_50U_, see Figure 1C). When no CDM is present in the head capsule, C_50_ is approximately doubled in the adapted neuron compared with the un-adapted neuron (gray symbols, Figure 3A). After CDM application, the adaptation induced C_50_ increase is significantly larger after all adapting temporal frequencies (Figure 3A, compare gray and black symbols). When no CDM is applied, the size of the contrast gain reduction does not depend on the temporal frequency of the adapting stimulus (Figure 3A, gray symbols). However, the adaptation-induced C_50_ increase in the presence of CDM shows clear frequency dependence (Figure 3A, compare gray and black symbols, Two-Way repeated measures ANOVA, interaction of frequency and pharmacological state, *p* < 0.05).

The local, motion-specific, output range reduction reduces the adapted HSN neuron's output gain, even after normalizing for the after-potential (Figure 1C, and Harris et al., 2000). The un-adapted output range did not change after application of CDM (11.9 ± 2.9 mV and 11.8 ± 2.7 mV, respectively, see adapting frequency “0” in Figure 3B). Our data show a small, but non-significant output range reduction in the absence of CDM (Figure 3B, gray symbols). When CDM is applied the small output range reduction is potentially even smaller, but there is no significant difference between the two conditions (with and without CDM, Figure 3B, paired *t*-test).

The after-potential is clearly visible in the contrast response functions after adapting at 20 Hz, with or without CDM (Figure 2). The after-potential is present after adaptation at all temporal frequencies (Figure 3C). Previous work showed that the magnitude of the after-potential depends on the strength of the adapting stimulus (Harris et al., 2000; Kurtz et al., 2009b), which our data confirm: there is a larger after-potential after the stronger depolarizing adapting stimuli at 7.5 and 12.5 Hz (Figure 3C, *p* < 0.05, compared with 2.5 and 20 Hz conditions). However, there is no effect of CDM on the size of the after-potential (Figure 3C, compare black and gray symbols, paired *t*-tests, n.s.).

### CDM affects the un-adapted membrane conductance

**Figure 4:**
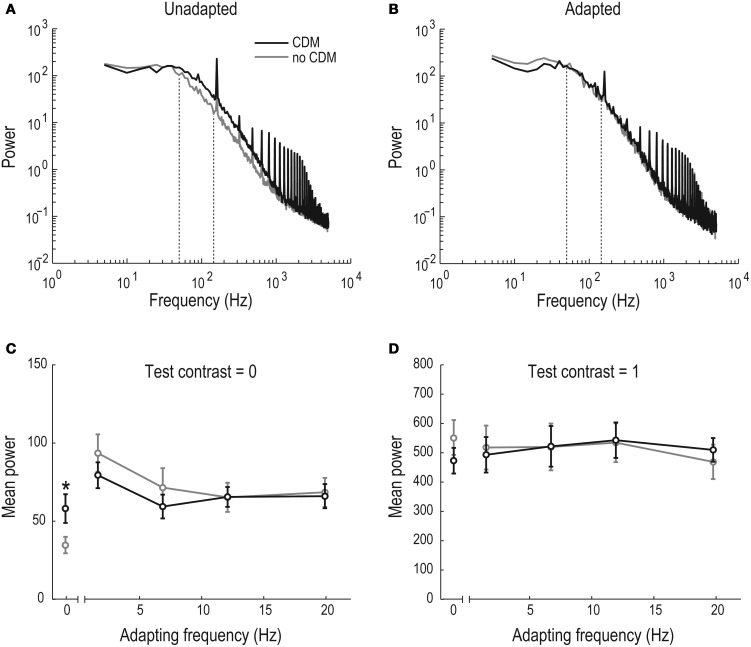
**CDM-induced increase of membrane potential power spectral density. (A)** The power spectral density of the HSN membrane potential in the unadapted and unstimulated neuron (*N* = 11). Between 50 and 1000 Hz the spectral power is increased in the CDM condition (black). Dashed lines indicate the window for quantitative analysis in panels **(C)** and **(D)**. The peaks at 160 Hz and its multiples are caused by our visual display. **(B)** The power spectral density of the HSN membrane potential in the adapted, but unstimulated neuron (*N* = 11). After the neuron was adapted for 1 s at 12.5 Hz the spectral power in the pre-CDM (gray) and CDM conditions (black) overlap. Dashed lines indicate the window for quantitative analysis in panels **(C)** and **(D)**. **(C)** Averaged power between 50–150 Hz as a function of adapting frequency, 100–300 ms post-stimulus onset, when the display was at mid-luminance. The significant separation (asterisk [^*^], *p* < 0.05) of the un-adapted power between the pre- and post-CDM conditions has disappeared after adaptation (*N* = 11). **(D)** Same as in panel **(C)**, but using a test contrast of 1.0. However, note the different scale of the y-axis (*N* = 11).

Maimon et al. (2010) showed that flight decreases the membrane resistance of un-stimulated *Drosophila* LPTCs compared with the resistance during rest. We investigated the presence of this effect after CDM application in *Eristalis* and the persistence of the conductance increase during and after stimulation of the neuron, using power spectrum analysis of the membrane potential. The power spectrum of the unadapted membrane potential, i.e., before any visual stimulation, shows a clear separation before (gray) and after (black) CDM application between ~50–1000 Hz (Figure 4A). This separation disappears after motion adaptation at 12.5 Hz, when the screen is at mid-luminance (contrast = 0, Figure 4B).

For further quantitative analysis of the membrane conductance we measured the mean power between 50–150 Hz 100–300 ms post-stimulus onset. This quantification confirms that the mean CDM-induced power increase (Figure 4A) is significant in the un-adapted neuron (“0” adapting frequency, Figure 4C). If we hypothesize that excitatory transmission underlies the CDM-induced reduced membrane resistance in the unadapted, unstimulated neuron (as seen in Figure 4A), this transmission would be likely to be reduced after adaptation, since the presynaptic elements would then be subject to a rebound-hyperpolarization. Indeed, following adaptation, when no synaptic transmission takes place, no CDM-induced conductance increase is generated in the adapted LPTC, but the power spectra before and after CDM application overlap (Figures 4B,C).

If the unadapted membrane resistance is reduced (Figure 4A) via increased excitatory synaptic activity, saturation of the synapses should lead to a smaller increase of the CDM-induced membrane conductance. To investigate this prediction we quantified the mean spectral power (50–150 Hz, 100–300 ms) in response to a test stimulus contrast of 1.0, before and after adaptation. Indeed, the CDM-induced increase of the mean power that we observed in the un-stimulated neuron (Figure 4A) disappears when it is strongly stimulated: there is no difference between the power spectral density with or without CDM in the stimulated, un-adapted (“0” adapting frequency, Figure 4D) or adapted neuron (Figure 4D). This supports the hypothesis that activity-induced increases in membrane conductances are caused by an increased baseline excitatory synaptic input, which decreases as synaptic transmission is saturated.

### CDM decreases the un-adapted latency

**Figure 5:**
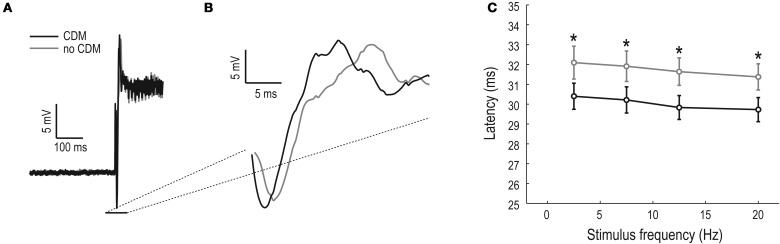
**CDM-induced decrease of the unadapted neural latency. (A)** Average response to an adapting stimulus moving at 7.5 Hz, in the preferred direction (*N* = 11, *n* > 200). **(B)** A magnification of the response onset, showing a clear separation of the responses between the pre-CDM (gray) and CDM conditions (black). **(C)** Response latency as a function of temporal frequency of the stimulus (0.1 cpd, contrast = 0.95, asterisks (^*^) indicate significant differences between pre-CDM and post-CDM, with significance allocated to *p* < 0.05, *N* = 10).

The data in Figure 4A show that the membrane resistance is reduced in the unadapted and unstimulated HS neuron. A reduced membrane resistance should lead to a reduction of the membrane time constant, which in effect generates a faster response. To investigate this hypothesis we look at the responses to a full-screen sinusoidal grating moving at 7.5 Hz, which show a definite difference between the response onset in the no-CDM and the CDM cases (Figures 5A,B). We quantified the unadapted response latencies to four temporal frequencies, and find that the neurons do respond significantly faster after CDM application (at all temporal frequencies, Figure 5, paired *t*-tests). This effect has previously been observed in spiking LPTCs in blowflies (Longden and Krapp, 2009).

The data shown in Figure 5 show the response delay in the unstimulated and unadapted neuron. This corresponds to the power spectrum shown in Figure 4A (quantified in the “0” condition, Figure 4C), which showed a clear separation between the no-CDM and the CDM state. We cannot quantify the adapted latency: since the second test stimulus immediately follows the adapting stimulus, this makes it impossible to determine the response threshold. However, since the power spectrums in the adapted neurons do not differ between the no-CDM and the CDM case (Figure 4B), it is unlikely that the CDM treated flies would respond faster in the adapted and/or strongly stimulated state.

## Discussion

In this paper we describe the responses of HSN neurons to test-adapt-test experiments. We used four different adapting frequencies, and we describe the change that adaptation undergoes when the octopamine receptor agonist CDM is applied to the fly's lobula plate. We found two effects of CDM, one working pre- and one postsynaptically, i.e., upstream of and within the LPTC itself, respectively. First, we found a CDM-induced, EMD linked modification of the contrast gain reduction, from temporal frequency independent to frequency dependent. Second, we found a CDM-induced, post-EMD increase of the power spectrum of the unadapted, unstimulated membrane potential, indicating decreased input resistance of HSN. This effect disappears during and after stimulation. Our findings thus support the suggestion (Jung et al., 2011) that temporal frequency specific effects of CDM or physical activity are generated within the EMDs, presynaptic of the LPTCs. Second, the decreased input resistance reported by Maimon et al. (2010) is unlikely to play a role in adaptation-linked effects of CDM, since this non-EMD effect is abolished by motion stimuli.

### Contrast gain reduction and an adaptive temporal delay filter

By using a test-adapt-test protocol we have here separated three different motion adaptation components to show that only one of these, the contrast gain reduction, is clearly affected by the application of CDM (Figure 3). Since contrast gain reduction is local (Nordström and O'Carroll, 2009) and fast (Nordström et al., 2011), it is likely to originate presynaptically of the LPTC, i.e., within the EMD (Figure 1D), suggesting that the adaptive effects we see after CDM-application are generated upstream of the LPTCs from which we record.

The LPTC velocity optimum of physically active, or CDM-treated, flies shifts to higher velocities, which can been modeled with altered time constants of the EMD's temporal filters (Chiappe et al., 2010; Jung et al., 2011). Whereas our data show that the temporal frequency dependent effects of CDM act presynaptically, we cannot determine its precise location. We can, however, speculate that it is located very early in the EMD, before the actual computation of motion in the multiplication stage of the model (Figure 1D), since contrast gain reduction is also mildly recruited by flicker, a non-motion visual stimulus (Harris et al., 2000). This would suggest that it is recruited before the medullary T4 and T5 neurons, since these are necessary for the transmission of motion information, but not of flicker stimuli (Schnell et al., 2012).

### CDM driven reduction of the input resistance in the un-stimulated neuron

Maimon et al. (2010) showed that the input resistance of *Drosophila* LPTCs decreases during flight and suggested that this effect is caused by increased excitatory synaptic input. This claim is supported by the observed increased resting membrane potential during flight (Maimon et al., 2010), or increased spontaneous activity generated during haltere movement (Rosner et al., 2010) or after CDM application (Longden and Krapp, 2009, 2010), of different LPTCs.

In previous work a neuron's input resistance has been measured directly (Kurtz, 2007), or by quantifying the SD of the neuron's baseline membrane potential before, during and after flight (Maimon et al., 2010; Rosner et al., 2010). We here use a power spectrum analysis, since increased activity at specific frequency bands in the membrane voltage power spectrum can be correlated with increased activity of specific processes in a neuron's membrane, e.g., the amplification of high-frequency fluctuations by voltage gated channels (Haag and Borst, 1996, 1998; Nordström and O'Carroll, 2009), or synaptic input mediated by different types of receptors (Destexhe and Rudolph, 2004; Rudolph et al., 2004). We found that there is an input resistance decrease of the HSN membrane when CDM is applied (Figures 4A,C) and that this is a non-EMD effect, since stimulating the EMDs with motion abolishes the CDM induced resistance changes (Figures 4B,D). This also indicates that the CDM-increased conductance cannot be responsible for any adaptive effects, since presynaptic activation of EMDs, and thus motion adaptation, abolished the conductance difference for at least up to 300 ms post-adaptation, corresponding to the end of our analysis window (Figures 4B,C). This is supported by our observation that the after-potential, which is generated within the LPTCs themselves (Figure 1D, and see Nordström and O'Carroll, 2009), is unaffected by CDM application (Figure 3C).

### Data analysis and its pitfalls

In recent work on the effect of behavioral state on LPTC motion adaptation, the effect was quantified at the end of a several seconds long continuous stimulus (Longden and Krapp, 2010; Jung et al., 2011). However, here we quantified adaptation by measuring the responses to a distinct test stimulus with a different temporal frequency than the adapting stimulus. Whereas the experimental protocols are not directly comparable, the test-adapt-test stimulus allowed us to extract different components of adaptation. In future work it could be interesting to quantify CDM-induced effects after longer adapting durations.

We used a Weibull fit to extract the C_50_. Using curve fitting to extract data points obviously depends on the quality of the fit. Here we used a simplex method (Lagarias et al., 1998), which is fairly established as a reliable fitting tool (e.g., Vaina and Dumoulin, 2011; Saleem et al., 2012). Importantly, however, we reach the same conclusions using other curve fitting methods, such as a least squares fit (data not shown). Furthermore, in recent work (Harris et al., 2000; Nordström and O'Carroll, 2009) we showed that quantifying C_50_ or other measures of contrast sensitivity at much lower contrasts, using either Weibull functions or other curve fitting techniques, all lead to the same qualitative conclusion. It is therefore unlikely that the observed CDM-induced increase of C_50_ is an artifact of our analysis.

We quantified responses 100–300 ms post-test stimulus onset. This window has been used in several previous publications (e.g., Harris et al., 2000; Kalb et al., 2008; Nordström et al., 2011), but some of the earliest effects of motion adaptation may not be visible this far into the response. For example, we showed that CDM has a clear effect on the initial response delay, only 30 ms after stimulus onset (Figure 5). In previous work we adapted in both the preferred and the non-preferred direction, and subtracted the responses from each other, to be able to quantify adapted responses much earlier (Nordström et al., 2011). Importantly, the key conclusions from such a subtractive analysis do not differ from the data analyzed 100–300 ms post-test stimulus onset after adapting in only one direction.

In our power spectrum analyses we quantified the mean power at 50–150 Hz (Figure 4). Importantly, we saw a CDM-induced increase of the power spectrum across a broad range of frequencies (50–1000 Hz, Figure 4A), making it unlikely that the effects we show depend on the specific analysis window. This is supported by the observation that the power spectrum in the no-CDM and CDM cases perfectly overlap across all these frequencies in the adapted neuron (50–1000 Hz, Figure 4B).

### Neuroethology of adaptation

Considering that LPTCs are involved in the detection of widefield optic flow, such as that generated by ego-motion, it seems physiologically relevant to change the coding of motion according to behavioral state. For example, we found that the reduction of the LPTC membrane resistance leads to an increase of response speed (Figure 5), which would be very useful for accurate maneuvering at high speeds, by allowing higher temporal resolution and lower response delays. However, during rest, fast changes in the membrane potential would lead to unnecessarily high metabolic costs (Laughlin, 2001), since every influx of Na^+^, Ca^2+^, or efflux of K^+^, must be compensated for by ATPase-mediated pumping of these ions against their gradient (Skou, 1957), to maintain the resting potential and resting ionic distribution (Hodgkin and Huxley, 1952).

It is likely that evolution has worked to minimize the metabolic costs associated with neural signaling (Niven and Laughlin, 2008), by reducing response amplitudes to prolonged stimulation, while maintaining sensitivity to relative changes (Maddess and Laughlin, 1985; Kurtz et al., 2009a). During rest, the only type of motion that may carry physiologically relevant information is found in transient motion impulses. Signaling levels to prolonged visual motion are indeed reduced in immobilized animals (see e.g., Maddess and Laughlin, 1985; Clifford and Langley, 1996; Harris et al., 2000; Kurtz et al., 2000; Kalb et al., 2008), whereas transient pulses remain reliably encoded (Maddess and Laughlin, 1985; Kurtz et al., 2009a).

When the animal is physically active, however, even continuous optic flow carries valuable information about flight course and this must therefore be properly encoded. The responses to continuous motion thus remain elevated compared with animals at rest (Longden and Krapp, 2010; Jung et al., 2011). Importantly, *Eristalis* can be physically active without generating large-field optic flow: hoverflies are characterized by their ability to hover stationary mid-air (Fitzpatrick and Wellington, 1982), thus reducing all background optic flow. In this case, the only relative motion is generated by e.g., other flying insects, or by branches moving in the wind. Transient motion impulses may therefore carry higher behavioral relevance to a hoverfly than it does to a blowfly.

## Conclusion

Motion adaptation can be broken down into separate components, generated within the neuron itself, and by presynaptic mechanisms. By using a test-adapt-test protocol we have here showed that mechanisms working presynaptically of the LPTCs affect adaptation during physical activity or CDM stimulation. Furthermore, since the effect of CDM on the neuron's conductance disappears when it is stimulated and adapted, this is likely to have minimal impact on adaptation. We thus find it likely that whereas CDM operates at several stages of the motion vision pathway, the effect it has on motion adaptation is generated within the EMDs, and not within the LPTCs themselves.

### Conflict of interest statement

The authors declare that the research was conducted in the absence of any commercial or financial relationships that could be construed as a potential conflict of interest.

## Abbreviations

CDM: chlordimeform
cpd: cycles per degree
EMD: elementary motion detector
HSN: horizontal system north
LPTC: lobula plate tangential cell
n.s: not significant.
